# The first host plant dataset of Curculionidae Scolytinae of the world: miscellaneous Tribes

**DOI:** 10.1038/s41597-024-02977-y

**Published:** 2024-01-24

**Authors:** Matteo Marchioro, Davide Vallotto, Enrico Ruzzier, Laura Besana, Michele Rossini, Giacomo Ortis, Massimo Faccoli, Isabel Martinez-Sañudo

**Affiliations:** 1 Department of Agronomy, Food, Natural Resources, Animals and Environment (DAFNAE), Università degli Studi di Padova, via dell’Università 16, 35020 Legnaro, Italy; 2 Department of Science, Università Roma Tre, viale G. Marconi 446, 00146 Rome, Italy; 3 Department of Biotechnology, Università di Verona, strada le Grazie 15, 37134 Verona, Italy

**Keywords:** Entomology, Forestry

## Abstract

Tribes Coriacephilini, Corthylini, Cryphalini, Ernoporini, Trypophloeini, Xyloctonini, and Xyloterini (Coleoptera: Curculionidae; Scolytinae) include spermophagous, phloeophagous, and xylomycetophagous species. Besides direct damage caused by burrowing into host plant tissues, some species are vectors of aggressive pathogens causing plant dieback and death, with consequent economic and ecological relevance. The international trade in plants and wood products is one of the main pathways for the introduction of non-native species worldwide. In this context, data availability on host plants and their economic uses is essential in pest risk assessment and for planning effective detection and monitoring strategies against invasive species. This paper provides a complete and updated list of host plants, with economic categorization, for 2139 scolytine species.

## Background & Summary

The Scolytinae (Coleoptera: Curculionidae) is a highly diverse subfamily of weevils inhabiting all regions of the world, except Antarctica;^1^ this group, which monophyly is still disputed, unites taxa characterized by their adaptation to bore into plant tissues at the adult stage^2,3^. Scolytinae apparently evolved during the mid-Cretaceous in parallel with the radiation of Spermatophyta^4^. Although scolytine beetles are primarily xylophagous, they are adapted to feed and develop on all plant parts, including twigs, leaf petioles, grass stems, seeds, and fruits^5^. In addition, Scolytinae are capable of developing not only on stressed, dying, or dead plants but also on healthy ones, especially thanks to symbiotic fungi, which can improve to the detoxification of host tissues or debilitation of the host plant^6^.

Biological invasions represent a serious issue for both the economy and the environment^7–9^. Among invasive Coleoptera, bark and ambrosia beetles (*i.e*., Scolytinae) are the most commonly intercepted and introduced worldwide^10,11^. In fact, given their bio-ecological features, they are easily transported inside wood packaging materials, timber, live plants, and wood products, and can overcome adverse conditions during travel and elude phytosanitary controls^12,13^.

Phytosanitary risk assessment regarding scolytine beetles is substantially based on knowledge of the species’ host plants^14^, allowing the development of effective monitoring and detection strategies^15–18^. For this reason, quick access to this relevant information (*i.e*., Scolytinae host plants) is crucial^14^. However, to date, this information is spread over a huge amount of papers, catalogs and books, not always easily accessible

The aim of this contribution is to present the most updated information available in the literature about the host plants recorded for scolytines of selected tribes. After the first paper devoted to the host plants of tribe Xyleborini LeConte, 1876^19^, the aim of this new contribution is to summarize the host plant records for the tribes Coriacephilini Johnson, 2020, Corthylini LeConte, 1876, Cryphalini Lindemann, 1877, Ernoporini Nüsslin, 1911, Trypophloeini Nüsslin, 1911, Xyloctonini Eichhoff, 1878 and Xyloterini LeConte, 1876. These tribes are treated together according to their recent reclassification^20–22^ and extensive systematic review^23^, which separated them from the former tribe Cryphalini (*sensu antiquo*)^24^.

These tribes include species of relevant phytosanitary interest such as *Hypothenemus eruditus* (Trypophloeini), an extremely polyphagous species with hundreds of host plants^25–27^ and widely established worldwide^27–29^; *Hypothenemus hampei* (Trypophloeini), which is a polyphagous and widespread scolytid beetle, a major pest on *Coffea* spp.^30–32^; and *Pityophthorus juglandis* (Corthylini), an invasive bark beetle^33–35^ vector of *Geosmithia morbida* Kolarík *et al*.^36^, an aggressive pathogen causing thousand canker disease on walnut trees (*Juglans* spp.)^36^.

## Methods

### Host plant definition

A specific definition of host plant was used in the compilation of this database, in accordance with Ruzzier *et al*.^19^. Only records of scolytine species observed boring inside any plant part or tissue (both at adult and larval stage) were accepted as a potential scolytine-plant association. Records derived from trapping or other observations of occurrence in plantations (including in monocultures) were considered unreliable and therefore not included in the dataset. According to this definition we have not distinguished between primary or secondary host plants, as well as reproductive hosts versus occasional hosts. This categorization, which may be important in other phytosanitary areas, is not essential from the point of view of preventing new introductions: in fact, even a secondary or occasional host can act as a vector of introduction. In addition, several species exhibit a high level of ecological stochasticity, which may lead them to attack either the same host trees with different intensity, phylogenetically related species or completely different plants^37–39^. For this reason, it was preferred to adopt a broader and more precautionary definition of “host”.

### Data collection

We generated the dataset using the scolytine species listed in the most recent and updated catalogs. Thus, this checklist was initially based on the Wood & Bright catalog^26^ and following supplements^28,40–42^, and integrated with data obtained from new taxonomic papers (*e.g*.^23^). The current version of the database includes all members of the tribes Coriacephilini, Corthylini, Cryphalini, Ernoporini, Trypophloeini, Xyloctonini, and Xyloterini (*sensu*^23^), described before February 28th 2023.

The research of host plants was conducted through a systematic search, treating individual scolytine species one at a time. The search included both valid species names and their synonyms. The research was performed in Google Scholar and Google using selected keywords such as species name of the scolytine (*e.g*., “*Hypothenemus eruditus*”), also in combination with other keywords such as “host”, “pest” and with the Boolean operators “AND”, “OR”, “NOT”, and double inverted commas for specific word combinations. Furthermore, data collection was integrated through the extensive revision of reports (annual, research, technical, project, etc.), working papers, government documents, evaluations, websites, and online resources^27,43^, as well as books, catalogs and manuals. Multilingual sources were consulted (e.g. English, French, German, Italian, Portuguese, Spanish), including idioms (e.g. Chinese, Japanese, Russian), and using, where necessary, instant translation sites (DeepL or Google translate). In order to guarantee as precise and exhaustive as possible result, a host research was made for each scolytine species until the search no longer produced new records.

Plant taxonomy adopted in the dataset was based on the information available from the “Plants of The World Online” database (POWO)^44^, whereas the economic uses of plant species were based on the “U.S. National Plant Germplasm System” database^45^. Economic use of plant species was organized in three categories: 1) Food and pharmaceutical (human and animal food, plants used in medicine - also folklore -, materials for food, chemical/pharmaceutical industry, etno-biological use, and bee plants); 2) Material (plants used as materials, furniture and fuel); 3) Environmental (plants of ornamental, shade and shelter, or domestic usage). The classification and names of economic uses were modified with respect to the one presented in the previous work on Xyleborini^19^, so any future updates will follow this new classification.

The references included in the database were both the most relevant, the most updated and those specifically referring to a determined species and its hosts. In the case of multiple references referring to the same species or reporting the same information, only one, and generally the first recovered or the most exhaustive, was selected and included in the database. Uncertain or imprecise records, including records using vernacular or local names, which could not be traced back to a reliable host species, were not included.

## Data Records

The database for the host plants of the world species of these tribes is available on Zenodo with the original database in XLSX format (i.e. “Complete_dataset_former_Cryphalini.xlsx”)^46^; the reference list is included in the same file as a different spreadsheet (“References”). The database is organized in four sheets. The first (*i.e*., Versions) summarizes all the updates introduced with respect to the previous version of the dataset. The second (*i.e*., Dataset) is organized in nine columns as follows: “Tribe” and “Species” include the taxonomic information on the scolytine beetles; “Host Family”, “Host Genus”, and “Host Species” include information on the plants, while “Reference” is where the beetle-plant association is reported. The last three columns (*i.e*., “Food and pharmaceutical”, “Material”, and “Environmental”) refers to the economic categories. In the “References” column, entries left blank refer to those Scolytinae species whose hosts remain unknown, both for lacking of data and publications specifically indicating the missing information (in this second case, the reference was not given). In the dataset, scolytines are sorted alphabetically by tribe, genus and species. Plant family and genus records do not imply that a specific scolytine species feeds on all the plants belonging to that category, but instead that this data is the most specific/detailed information available in the reviewed literature, thereby suggesting that a determined scolytine species feeds on at least one plant species belonging to that specific family/genus. The third sheet (*i.e*., Economic Uses) provides the alphabetically ordered plant list with associated economic usages. The fourth (*i.e*., References) lists all the references (in alphabetical order) used in the creation of the second sheet “Dataset”.

The database will be periodically updated with new versions (namely Version 1.0 onwards); the latest and most updated database will be the first to access via the DOI provided here, however previous versions of the same file will also remain available in the repository. The first version (1.0) provides information for 2,139 species of Scolytinae, of which 936 have no host records available. Of the 1,203 species in which the host is known, 913 species have their host known at species level, while for 290 our knowledge of the host is limited to the family or genus. The dataset includes records for 1,405 plant species, distributed among 151 families and 729 genera; 835 plant species belong to at least one of the three economic categories considered.

## Technical Validation

All host records included in the database are based on articles published in scientific journals, books, reports and databases managed by leading experts on scolytine beetles (e.g. Atkinson database: Bark and Ambrosia Beetles of the Americas);^27^ therefore, we have confidence in their accuracy, frequently guaranteed by the peer-review process. In addition, we included host records recovered from databases managed by international phytosanitary agencies (e.g. CABI^43^ and EPPO^47^), aware that in some cases data provided may be considered uncertain or not counter-validated by publications.

As already specified in materials and methods, to standardize and harmonize the information we critically reviewed all the data collected, keeping only that related to the species whose relationship with the host plant could be recognized unequivocally; for this reason, we have excluded all possible cases that do not fall within the standards defined in the materials and methods section. Each record in the dataset is associated with a bibliographic reference, allowing users to assess the validity of the record, and reuse the data. We listed the references cited in the database, making it possible for users to access the original sources.

Scolytine species taxonomy is standardized following the International Code of Zoological Nomenclature (ICZN)^48^. The complete list of genera and species belonging to the target tribes was compiled using Bright’s catalog^28^, integrated with the latest publications and, finally, cross-validated by Andrew J. Johnson (University of Florida), an expert on these tribes. Plant taxonomy follows the International Code of Nomenclature for algae, fungi and plants^49^. Taxon names and authors, including subspecies, varieties and hybrids are consistent with those provided in the internationally recognized POWO database and the International Plant Names Index (IPNI)^50^.

Since listing host plants is a dynamic activity, especially for non-native and invasive species, and scolytine taxonomy is in continuous evolution, our aim is to keep updating the species list and host plant data starting from our direct research upon literature, as well as direct contribution from scolytine beetle researchers and stakeholders. Data will be corrected and updated if any errors or updates are reported to the first author (matteo.marchioro@unipd.it).

## Usage Notes

The data descriptor was peer reviewed in 2023 based on the data available on the platform at the time. Since this is a dynamic dataset, it may undergo changes or updates in the future.

## Data Availability

No custom code was used to generate or process the data described in the manuscript.
